# Selective inhibition of CBP/p300 HAT by A-485 results in suppression of lipogenesis and hepatic gluconeogenesis

**DOI:** 10.1038/s41419-020-02960-6

**Published:** 2020-09-11

**Authors:** Feiye Zhou, Qianqian Liu, Linlin Zhang, Qin Zhu, Shushu Wang, Kecheng Zhu, Ruyuan Deng, Yun Liu, Guoyue Yuan, Xiao Wang, Libin Zhou

**Affiliations:** 1 Shanghai Clinical Center for Endocrine and Metabolic Diseases, Department of Endocrine and Metabolic Diseases, Shanghai Institute of Endocrine and Metabolic Diseases, Ruijin Hospital, Shanghai Jiaotong University School of Medicine, Shanghai, 200025 China; 2 Department of Gastroenerology and Hepatology, Zhongshan Hospital, Fudan University, Shanghai, 200032 China; 3 Department of Endocrinology, Affiliated Hospital of Jiangsu University, Zhenjiang, Jiangsu 212001 China

**Keywords:** Endocrinology, Endocrine system and metabolic diseases

## Abstract

The histone acetyltransferases CREB-binding protein (CBP) and its paralogue p300 are transcriptional coactivators which are essential for a multitude of signaling pathways and energy homeostasis. However, the role of CBP/p300 HAT domain in regulating energy balance is still unclear. Here, C57BL/6 mice fed with either normal chow diet (NCD) or high-fat diet (HFD) were administrated with A-485, a recently reported selective inhibitor of CBP/p300 HAT activity for 1 week and the metabolic change was analyzed. The white adipose tissue (WAT) weight and adipocyte size were reduced in A-485-administrated mice, with decreased expressions of lipogenic genes and transcriptional factors. In the liver of A-485-treated mice, the lipid content and lipogenic gene expressions were lowered while the binding of forkhead box O1 (FOXO1) to glucose-6-phosphatase (G6Pc) promoter was reduced, leading to decreased expression of G6Pc. In primary mouse hepatocytes, A-485 abolished cAMP-elicited mRNA expressions of key gluconeogenic enzymes and promoted FOXO1 protein degradation via increasing its ubiquitination. Thus, A-485 inhibits lipogenesis in WAT and liver as well as decreases hepatic glucose production via preventing FOXO1 acetylation, leading to its protein degradation through a proteasome-dependent pathway. The specific inhibition of CBP/p300 HAT will provide a novel therapeutic approach for metabolic diseases.

## Introduction

Protein acetylation is a key post-translational modification (PTM) in cellular regulation. With advances in mass spectrometric technologies, both histone and nonhistone have been proved to be targets of acetylation in nearly all intracellular compartments^1,2^. It has been demonstrated that protein acetylation may serve as a broad bridge between extracellular nutrient status and intracellular metabolic pathways in physiological and pathological processes^2^. Thus, control of acetylation has emerged as an attractive therapeutic strategy for several metabolic diseases^3,4^, which motivates intense drug discovery efforts in this area^5^. Previous researches have paid more attention to the effects of histone deacetylases on the regulation of energy homeostasis. However, the role of counter-regulators histone acetyltransferases (HATs) in metabolic processes is poorly understood.

As the KAT3 family of HATs^6^, CREB-binding protein (CBP) and p300 can acetylate lysine residues on histones to change chromatin structure and function, as well as lysine residues on nonhistone proteins to modulate their activities^7^. The early embryonic lethality observed in CBP/p300 knockout mice indicates the important role of CBP/p300 in normal development^8,9^. CBP and p300 are large proteins that contain several conserved domains, including NRID, CH1 (TAZ1), KIX, Bromodomain, PHD, HAT, ZZ, TAZ2, and NCBD^10^. The domains of CBP/p300 exhibit functional diversity by providing scaffolds for the interaction with their target regulators. HAT domain functions as an acetyllysine “writer” that acetylates target proteins and bromodomain functions as an acetyllysine “reader” that binds to target proteins^11^. It is still unclear whether CBP/p300 HAT domain is important for whole animal energy homeostasis while KIX domain and CH1 domain have been reported to participate in metabolic control^12,13^. It will be promising to develop a highly selective inhibitor of CBP/p300 HAT, so as to better understand the association of CBP/p300 HAT with overall metabolic signaling pathways.

Several HAT inhibitors are routinely used to probe epigenetic pathways, but most of them show poor selectivity in vitro^14,15^. The most recently reported CBP/p300 HAT inhibitor A-485 represents a significant step forward in the development of HAT chemical probes. A-485 can inhibit the acetylation activity of both CBP and p300 and is at least 1000-fold more potent than previously described cell-permeable tool compounds. As a drug-like HAT inhibitor, A-485 inhibited proliferation in lineage-specific tumor types, including several hematological malignancies and androgen receptor-positive prostate cancer^16^. It has been underscored for evaluating the clinical utility of A-485 in multiple human cancers, but its metabolic effect remains unclear. By giving mice 1 week administration of A-485, our study demonstrated in vivo metabolic effect of A-485 and provided insight into how CBP/p300 HAT maintained metabolic homeostasis, especially in white adipose tissue (WAT) and liver.

## Material and methods

### Animal experiment

Male C57BL/6 mice were purchased from Shanghai Slack Experimental Center. Mice were housed in a barrier facility with 12 h light/12 h dark cycles. The mice were intraperitoneally injected with A-485 (20 mg · kg^−1^ · day^−1^) for 1 week under either normal chow diet (NCD) or high-fat diet (HFD) condition. All animal protocols were reviewed and approved by the Animal Care Committee of Ruijin Hospital, Shanghai Jiaotong University School of Medicine.

Lean and fat mass were determined via EchoMRI in live. For glucose tolerance test (GTT), mice were intraperitoneally injected with 2 g/kg body weight of glucose after 16 h fasting. For pyruvate tolerance test (PTT), mice were intraperitoneally injected with 2 g/kg body weight of pyruvate after 16 h fasting. For insulin tolerance test (ITT), mice were intraperitoneally injected with 0.75 UI/kg body weight of insulin after 6 h fasting.

### Primary hepatocyte isolation and culture

Primary mouse hepatocytes were isolated from C57BL/6 mice by a two-step perfusion technique as described^17^. Cells were treated with 100 μM 8-Bromoadenosine 3′,5′-cyclic monophosphate (8-Br-cAMP, Sigma), 3 μM A-485 (MedChemExpress), 10 μM MG132 (Beyotime Biotechnology), or 10 μg/ml cycloheximide (CHX, Sigma).

### Primary white adipocyte differentiation

Stromal vascular (SV) cells isolated by collagenase digestion of minced inguinal WAT from C57BL/6 mice were plated onto a 6 cm tissue culture dish and cultured in 10% CO_2_ at 37 °C. For white adipocyte differentiation, SV cells were expanded in growth media containing DMEM/F12 and 10% FBS. At confluence, cells were exposed to growth media supplemented with 5 μg/ml insulin, 1 μM dexamethasone, and 0.5 mM isobutylmethyxanthine for 48 h, and then maintained in growth media containing 5 μg/ml insulin for 6 days. Cells were fully differentiated on day 8.

### Glucose production assay

Primary mouse hepatocytes were seeded into 24-well plates and pre-treated with 100 nM dexamethasone for 16 h. Then the medium was replaced with glucose production buffer consisting of glucose-free DMEM supplemented with 1 mM sodium pyruvate, 10 mM sodium lactate, and 0.25% BSA. After 24 h, the medium was collected for measuring glucose content by a colorimetric glucose assay kit (Applygen).

### Real-time quantitative PCR (RT-qPCR)

Total RNA was extracted from mouse tissues or primary hepatocytes using Trizol regent. To quantify the transcript abundance of genes of interest, RT-qPCR was performed with a SYBR Green Premix Ex Taq (Takara) in an Applied Biosystems 7300 Real-Time PCR machine (Applied Biosystems). The primer sequences used for RT-qPCR were listed in Supplementary Table 1.

### Western blotting and immunoprecipitation

Tissues or cells were homogenized in lysis buffer (Cell Signaling Technology). Blotted membrane was imaged with a LAS-4000 Super CCD Remote Control Science Imaging System (Fuji). Immunoprecipitation assays were performed by incubating protein lysates with indicated antibodies for 2 h and then with protein A/G-agarose beads (Santa Cruz) overnight at 4 °C. The immunoprecipitates were washed and eluted with SDS loading buffer. Then standard western blotting was followed. The antibodies were listed in Supplementary Table 2.

### Nuclear and cytoplasmic protein extraction

Nuclear and cytoplasmic protein was extracted from primary mouse hepatocytes by means of commercial protocol and reagents (Thermo scientific) as previously described^18^.

### Immunofluorescence staining

HEK293T cells expressing HA-tagged FOXO1 were fixed for 20 min in 10% formalin, permeabilized in 0.1% Triton X100 for 5 min, washed with PBS, and blocked in 5% BSA for 1 h. Cells were then incubated with anti-HA antibody (1:200) overnight at 4 °C and stained with FITC-labeled IgG (1:200, Jackson ImmunoResearch Laboratories). DAPI was added to stain cell nuclei. The cellular localization of HA-tagged FOXO1 was photographed and analyzed using a fluorescence microscope (Olympus BX51).

### Ubiquitylation assay

For FOXO1 ubiquitylation analysis, HepG2 cells were transfected with plasmids of Flag-ubiquitin and HA-FOXO1 as indicated. Protease inhibitor MG132 and A-485 were added 4 h before harvest. 36 h after transfection, cells were collected and lysed in 1% SDS buffer. Immunoprecipitation of lysed proteins was performed by adding anti-HA antibody and ubiquitinated FOXO1 was detected by immunoblotting with anti-Flag antibody.

### Chromatin immunoprecipitation (ChIP)

ChIP assay was performed by the ChIP kit (Millipore). Briefly, liver tissues from mice were incubated with 1% formaldehyde and homogenized in cell lysis buffer. The lysates were sonicated to yield chromatin fragments of 200–1000 bp. Chromatins were incubated and precipitated with antibody against FOXO1 or IgG. DNA pellets were analyzed by RT-qPCR by using primers directed to the glucose-6-phosphatase (G6Pc) promoter (Supplementary Table 3).

### Statistics

Data were expressed as mean ± SEM. Comparisons were performed using *ANOVA* for multiple groups or the Student’s *t* test for two groups. Statistical significance was established at *P* < 0.05.

## Results

### A-485 decreases body weight and fat mass in C57BL/6 mice

**Fig. 1:**
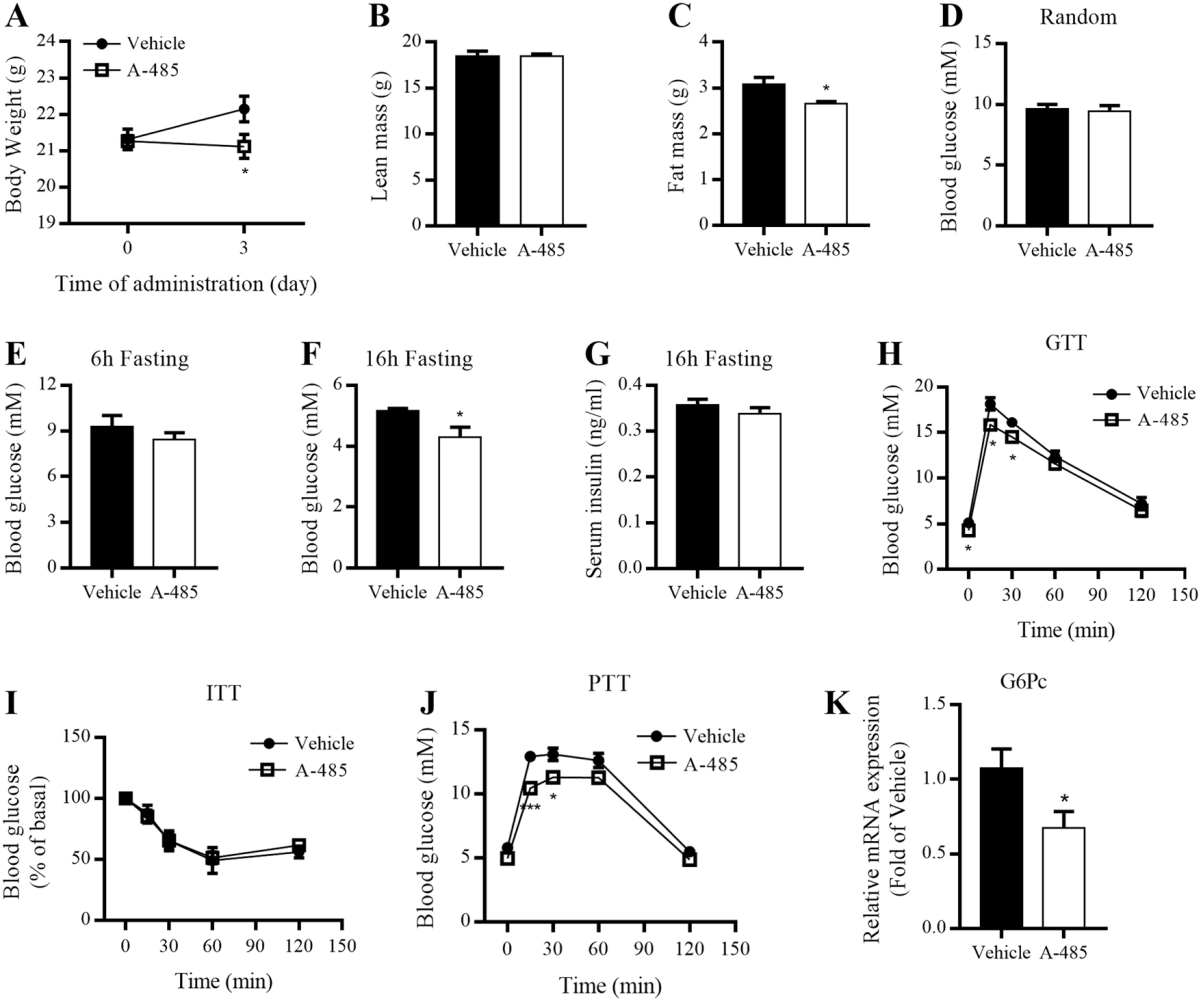
A-485 administration decreases body weight and fat mass of C57BL/6 mice. 8-week-old C57BL/6 mice were given A-485 (20 mg · kg^−1^ · day^−^^1^) daily by intraperitoneal injection for 7 days. **a** Body weight of mice treated with vehicle and A-485. **b**, **c** Total lean mass and fat mass. **d** Random blood glucose level. **e**, **f** Blood glucose levels after 6 and 16 h fasting. **g** Serum insulin level after 16 h fasting. **h** Blood glucose levels during intraperitoneal glucose tolerance tests (GTT) after 16 h fasting. **i** Blood glucose levels during insulin tolerance tests (ITT) after 6 h fasting. **j** Blood glucose levels during intraperitoneal pyruvate tolerance tests (PTT) after 16 h fasting. **k** G6Pc mRNA expression in the liver. Data are expressed as means ± SEM (*n* = 5–9). ^*^P < 0.05, ^***^P < 0.001 vs. vehicle group.

To investigate the in vivo metabolic properties of A-485, 8-week-old C57BL/6 mice fed with NCD were intraperitoneally administrated with A-485 (20 mg · kg^−1^ · day^−1^) for 1 week. A-485-administered mice displayed significantly lower body weight compared with vehicle-administered mice after treatment for 3 days (Fig. 1a). However, food intake was comparable between vehicle- and A-485-administered mice (Fig. S1a). No significant changes were observed for the oxygen consumption (VO_2_), carbon dioxide (VCO_2_) production, and respiratory exchange ratios (RER) in mice after A-485 administration (Fig. S1b–d). A-485 decreased total fat mass by ~14%, without affecting total lean mass (Fig. 1b, c). These results indicate that A-485-induced weight loss is attributable to decreased fat mass.

### A-485 treatment reduces hepatic glucose production in mice

We further assessed the effects of A-485 on glucose and lipid metabolism in C57BL/6 mice. No differences were observed in plasma total cholesterol and triglyceride levels between two groups (Fig. S1e, f), with comparable random and 6 h fasting blood glucose levels (Fig. 1d, e). However, when fasting was prolonged to 16 h, fasting blood glucose level was decreased in A-485-administered mice compared with control mice (Fig. 1f), without significant difference in fasting serum insulin (Fig. 1g). GTT revealed a mild decrease in blood glucose levels of A-485-administered mice 15 and 30 min after glucose loading (Fig. 1h), while ITT showed comparable insulin sensitivity between two groups (Fig. 1i). Moreover, the blood glucose levels were lower in A-485-administered mice than those in control mice at 15 and 30 min during PTT (Fig. 1j). Hepatic G6Pc mRNA expression was dramatically decreased in A-485-administered mice (Fig. 1k), suggesting that A-485-suppressed hepatic glucose production contributes to decreased blood glucose level.

### Metabolic action of A-485 on mice fed with HFD

**Fig. 2:**
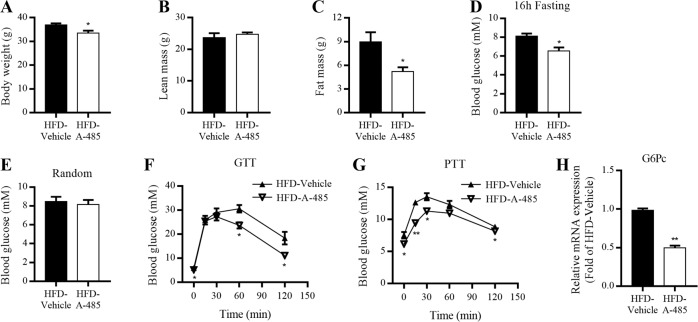
Metabolic effect of A-485 administration on mice fed with HFD. C57BL/6 mice fed with high-fat diet (HFD) for 14 weeks from 5 weeks of age were intraperitoneally injected with A-485 (20 mg · kg^−1^ · day^−^^1^) for 7 days. **a** Body weight of mice treated with vehicle and A-485 for 3 days. **b**, **c** Total lean mass and fat mass. **d** Blood glucose levels after 16 h fasting. **e** Random blood glucose levels. **f** Blood glucose levels during intraperitoneal glucose tolerance tests (GTT) after 16 h fasting. **g** Blood glucose levels during intraperitoneal pyruvate tolerance tests (PTT) after 16 h fasting. **h** G6Pc mRNA expression in the liver. Data are expressed as means ± SEM (*n* = 5). ^*^P < 0.05, ^**^P < 0.01 vs. HFD-vehicle group.

To further investigate the therapeutic potential of A-485 in obese animals, mice fed with HFD for 14 weeks were treated with A-485 and their metabolic profiles were evaluated using the same protocol as described in NCD-fed mice. Consistently, A-485 treatment decreased body weight and total fat mass without altering total lean mass (Fig. 2a–c). Compared with HFD-fed mice, A-485-administered mice also exhibited lower fasting blood glucose level (Fig. 2d), without significant change in random blood glucose level (Fig. 2e). Like in NCD-fed mice, A-485 administration improved glucose tolerance in mice fed with HFD (Fig. 2f) and considerably decreased blood glucose levels in the whole process of PTT (Fig. 2g). Furthermore, the mRNA expression of hepatic G6Pc in HFD-fed mice was also dramatically reduced by A-485 treatment (Fig. 2h).

### Effect of A-485 on the expression of lipid metabolism-related genes in adipose tissue

**Fig. 3:**
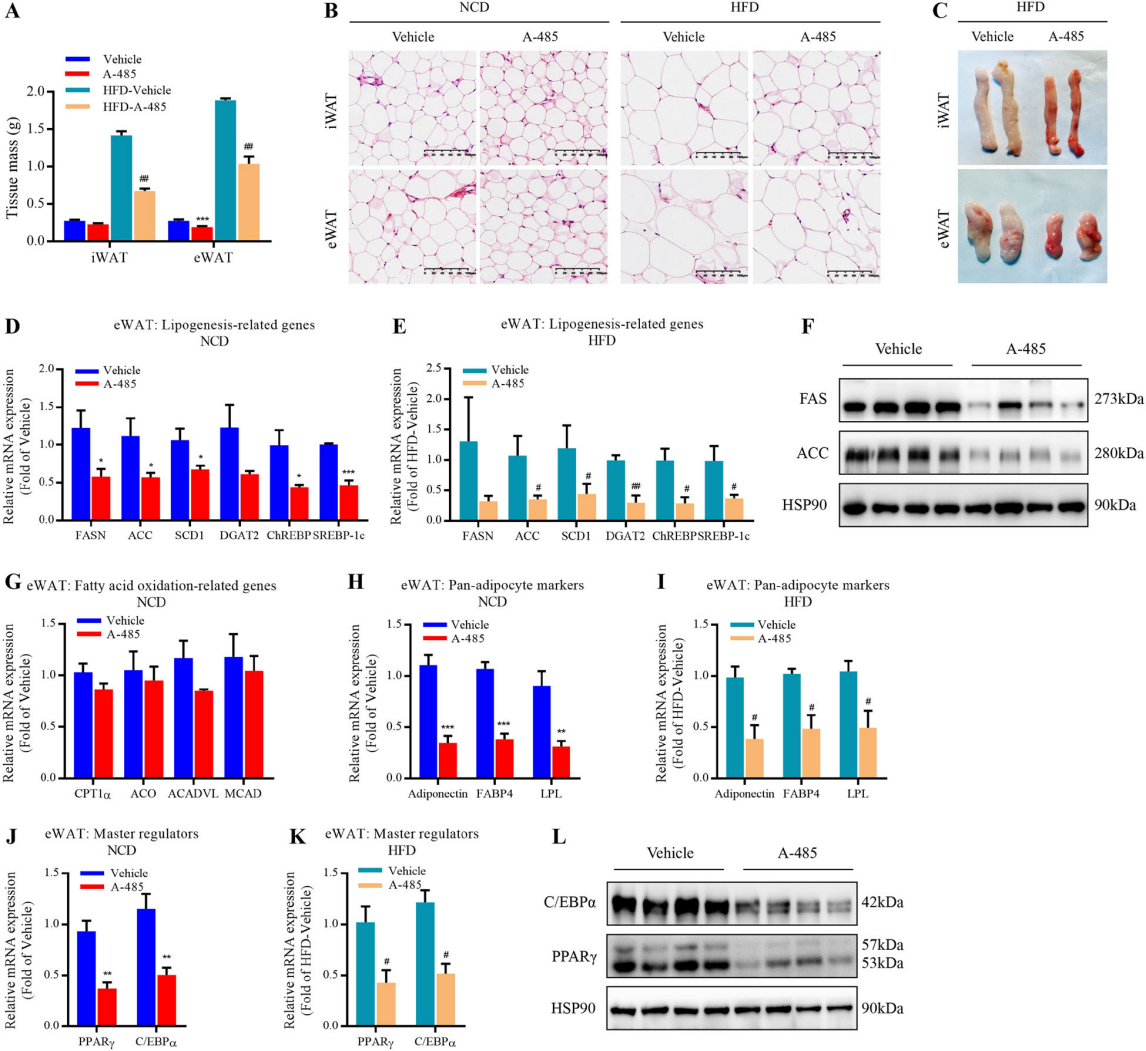
A-485 inhibits the expression of lipogenesis-related genes in white adipose tissue. **a** Weight of epididymal white adipose tissue (eWAT) and inguinal white adipose tissue (iWAT) from mice treated with vehicle and A-485 for 7 days under normal chow diet (NCD) and high-fat diet (HFD) conditions. **b** Hematoxylin-eosin staining of iWAT and eWAT (bar = 100 μm). **c** Morphology of the iWAT and eWAT from HFD-fed mice treated with vehicle and A-485. **d**, **e** mRNA expressions of lipogenesis-related genes in the eWAT from NCD- and HFD-fed mice. **f** Western blot analysis of fatty acid synthase (FAS) and acetyl-CoA carboxylase (ACC) protein levels in the eWAT from NCD-fed mice. **g** mRNA expressions of fatty acid oxidation-related genes in the eWAT from NCD-mice. **h**, **i** Adiponectin, FABP4, and LPL mRNA expressions in the eWAT from NCD- and HFD-fed mice. **j**, **k** PPARγ and C/EBPα mRNA expressions in the eWAT from NCD- and HFD-fed mice. **l** PPARγ and C/EBPα protein expressions in the eWAT from NCD-mice. Data are expressed as means ± SEM (*n* = 5–9). ^*^P < 0.05, ^**^P < 0.01, ^***^P < 0.001 vs. vehicle group. ^#^*P* < 0.05, ^##^*P* < 0.01 vs. HFD-vehicle group.

In parallel with A-485-mediated loss of fat mass, the drug did decrease the weight of epididymal white adipose tissue (eWAT) and inguinal white adipose tissue (iWAT) (Fig. 3a). As a result, the adipocyte size and gross size of WAT from A-485-administered mice were smaller than those of control mice, especially under HFD condition (Fig. 3b, c and Fig. S2a). We further detected the expressions of lipogenic genes in WAT of NCD- and HFD-fed mice. A-485 remarkably decreased the mRNA expressions of key lipogenesis genes and transcription factors, including FASN (encoding fatty acid synthase, FAS), acetyl-CoA carboxylase (ACC), acyl-CoA desaturase 1 (SCD1), diacylglycerol O-acyltransferase 2 (DGAT2), carbohydrate response element-binding protein (ChREBP), and sterol response element-binding protein 1c (SREBP-1c) (Fig. 3d, e and Fig. S2b, c). FAS and ACC protein expressions showed a similar result (Fig. 3f). However, there were no differences in the expressions of fatty acid oxidation-related genes such as carnitine palmitoyltransferase 1α, aconitate hydratase (ACO), medium-chain specific acyl-CoA dehydrogenase (MCAD), and very long-chain specific acyl-CoA dehydrogenase between two groups (Fig. 3g). Nor did the mRNA expression of classical brown adipocyte tissue marker gene uncoupling protein 1 (Fig. S2d). These results suggest that A-485 lowers fat mass mainly via repressing lipid biosynthesis in WAT, not by promoting lipid oxidation.

In addition, the mRNA levels of pan-adipocyte markers adiponectin, fatty acid-binding protein (FABP4), and lipoprotein lipase (LPL) were significantly decreased by A-485 in NCD- and HFD-fed mice (Fig. 3h, i and Fig. S2e,f). Furthermore, the mRNA and protein expressions of peroxisome proliferator-activated receptor gamma (PPARγ) and CCAAT/enhancer-binding protein alpha (C/EBPα), two master regulators of adipogenesis^19^, were also robustly downregulated (Fig. 3j–l and Fig. S2g,h).

In fully differentiated primary adipocytes from mice, the expressions of lipogenesis-related genes were markedly suppressed after A-485 incubation for 24 h (Fig. S3a–e), consistent with the in vivo results. These data suggest that the activity of CBP/p300 HAT domain is indispensable for maintaining normal adipogenesis and lipogenesis in WAT.

### A-485 represses hepatic lipogenesis

**Fig. 4:**
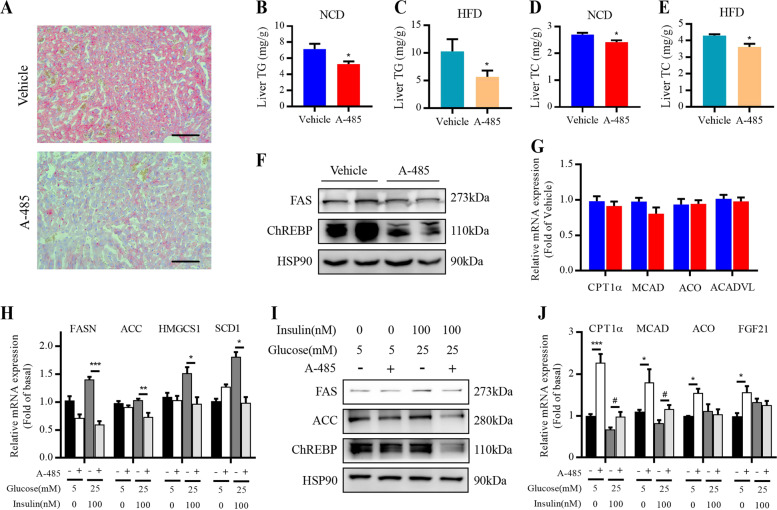
A-485 suppresses hepatic lipogenesis. **a** Oil red O staining of liver sections from normal chow diet (NCD)-fed mice treated with vehicle and A-485 for 7 days (bar = 100 μm). **b**, **c** Hepatic triglyceride of NCD-fed and high-fat diet (HFD)-fed mice. **d**, **e** Hepatic total cholesterol contents of NCD-fed and HFD-fed mice. **f** Fatty acid synthase (FAS) and carbohydrate-responsive element-binding protein (ChREBP) protein expressions in the liver from NCD-fed mice. **g** mRNA expressions of fatty acid oxidation-related genes in the liver from NCD-mice. **h**–**j** Primary mouse hepatocytes were incubated with 3 μM A-485 for 18 h in the presence of 0 or 100 nM insulin and 5 or 25 mM glucose. Lipogenesis- and fatty acid oxidation-related gene expressions were detected. Data are expressed as means ± SEM (*n* = 5–9). ^*^P < 0.05, ^**^P < 0.01, ^***^P < 0.001 vs. corresponding control group; ^#^*P* < 0.05.

CBP/p300 is involved in the regulation of hepatic lipogenic gene expression^20–22^. Therefore, we explored whether this function of CBP/p300 relies on the activity of its HAT domain. Examination of liver sections revealed reduced number of lipid droplet in A-485-administered mice compared to control mice (Fig. 4a), with significant decreases in hepatic triglyceride (Fig. 4b, c) and total cholesterol (Fig. 4d, e) contents. Consistently, A-485 lowered the protein levels of FAS and ChREBP (Fig. 4f). In consistent with the results in WAT, A-485 did not change the expressions of fatty acid oxidation-related genes in liver of mice (Fig. 4g). We further examined the impact of A-485 treatment on lipid metabolism in primary mouse hepatocytes. As shown in Fig. 4h, in the presence of 100 nM insulin and 25 mM glucose, A-485 significantly decreased the mRNA expressions of key lipogenesis and cholesterol synthesis genes, including FASN, ACC, SCD1, and HMG-CoA synthase (HMGCS1). Similarly, FAS, ACC, and ChREBP protein expressions exhibited remarkable decreases in A-485-treated hepatocytes (Fig. 4i). Though A-485 elevated the expression of fatty acid oxidation-related genes such as MCAD, ACO, CPT1α, and fibroblast growth factor 21 at basal status, the impact became weaker when high levels of insulin and glucose were present (Fig. 4j). These results indicate that A-485 reduces hepatic lipid accumulation mainly via repressing lipid biosynthesis.

### A-485 inhibits gluconeogenesis in primary mouse hepatocytes

**Fig. 5:**
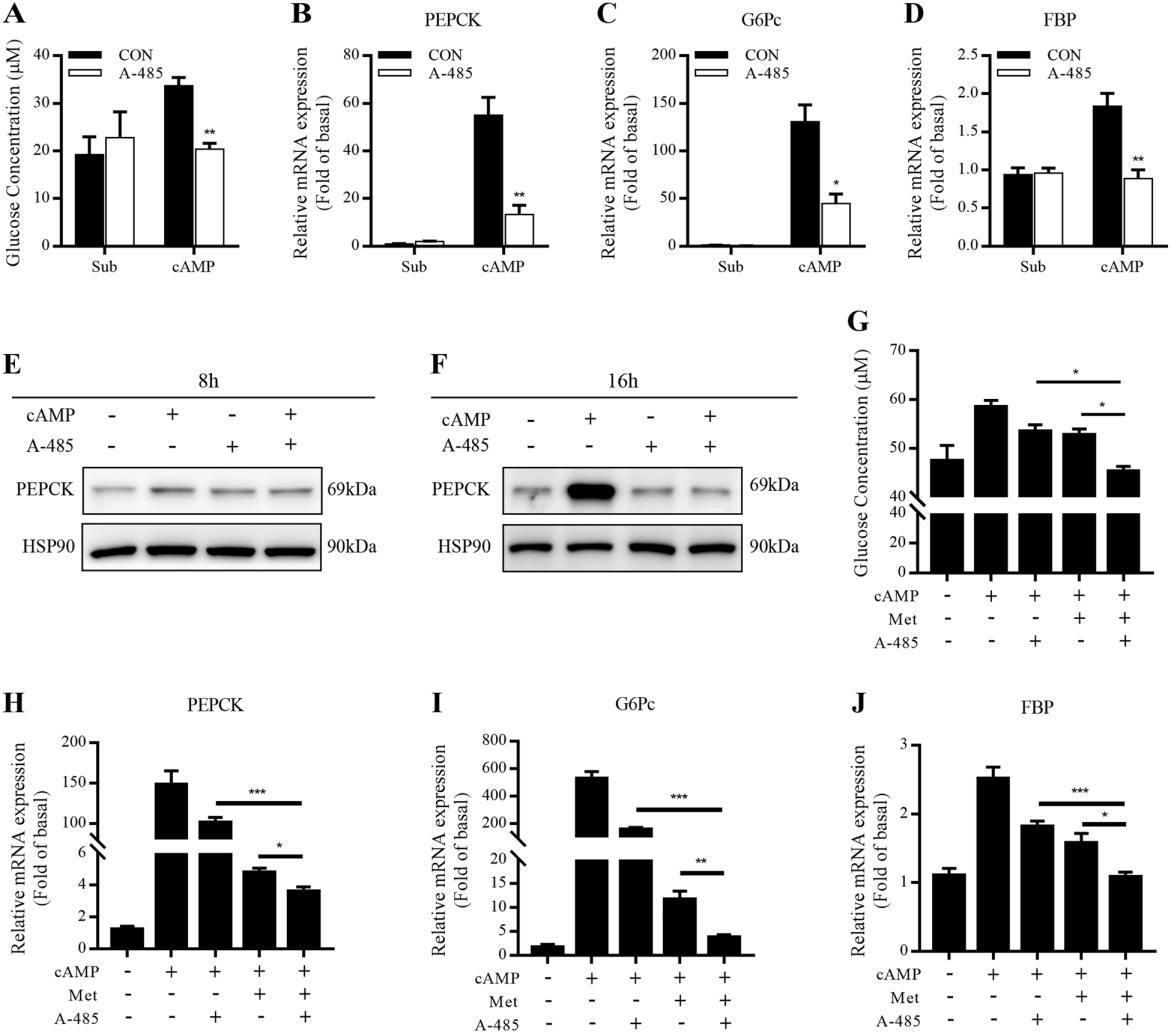
Effect of A-485 on gluconeogenesis in primary mouse hepatocytes. **a** Primary mouse hepatocytes were incubated with 3 μM A-485 and 100 μM 8-Br-cAMP in glucose-free DMEM containing gluconeogenic substrates (1 mM sodium pyruvate and 10 mM sodium lactate) for 24 h. The cell culture supernatants were collected for measuring glucose content. **b**–**d** mRNA expressions of gluconeogenic genes in primary mouse hepatocytes treated with 3 μM A-485 and 100 μM 8-Br-cAMP for 8 h. **e**, **f** Protein expression of PEPCK in primary mouse hepatocytes incubated 3 μM A-485 and 100 μM 8-Br-cAMP for 8 and 16 h. **g**–**j** Glucose production and gluconeogenic gene mRNA expressions in primary mouse hepatocytes treated with 3 μM A-485 and 2 mM metformin in the presence of 100 μM 8-Br-cAMP. Data are expressed as means ± SEM for three independent experiments. ^*^P < 0.05, ^**^P < 0.01, ^***^P < 0.001 vs. control (CON), metformin (Met) alone, or A-485 alone group.

Glucagon promotes hepatic gluconeogenesis through the cAMP-PKA signaling pathway^23^. In primary mouse hepatocytes, 8-Br-cAMP-stimulated glucose production was decreased after A-485 treatment for 24 h (Fig. 5a). cAMP-elicited mRNA expressions of three key gluconeogenic enzymes phosphoenolpyruvate carboxykinase (PEPCK), G6Pc, and fructose 1,6-bisphosphatase (FBP) were significantly abolished after A-485 treatment for 8 h (Fig. 5b–d), but PEPCK protein expression failed to be changed in the presence or absence of cAMP (Fig. 5e). Once the incubation time was prolonged to 16 h, cAMP-induced protein expression of PEPCK was dramatically decreased by A-485 (Fig. 5f). We incubated primary mouse hepatocytes with A-485 and antidiabetic drug metformin. Interestingly, two drugs exhibited a synergistic inhibitory effect on hepatic glucose production and expressions of three key gluconeogenic enzymes (Fig. 5g–j).

### Impact of A-485 on the expression of gluconeogenesis-related transcription factors

**Fig. 6:**
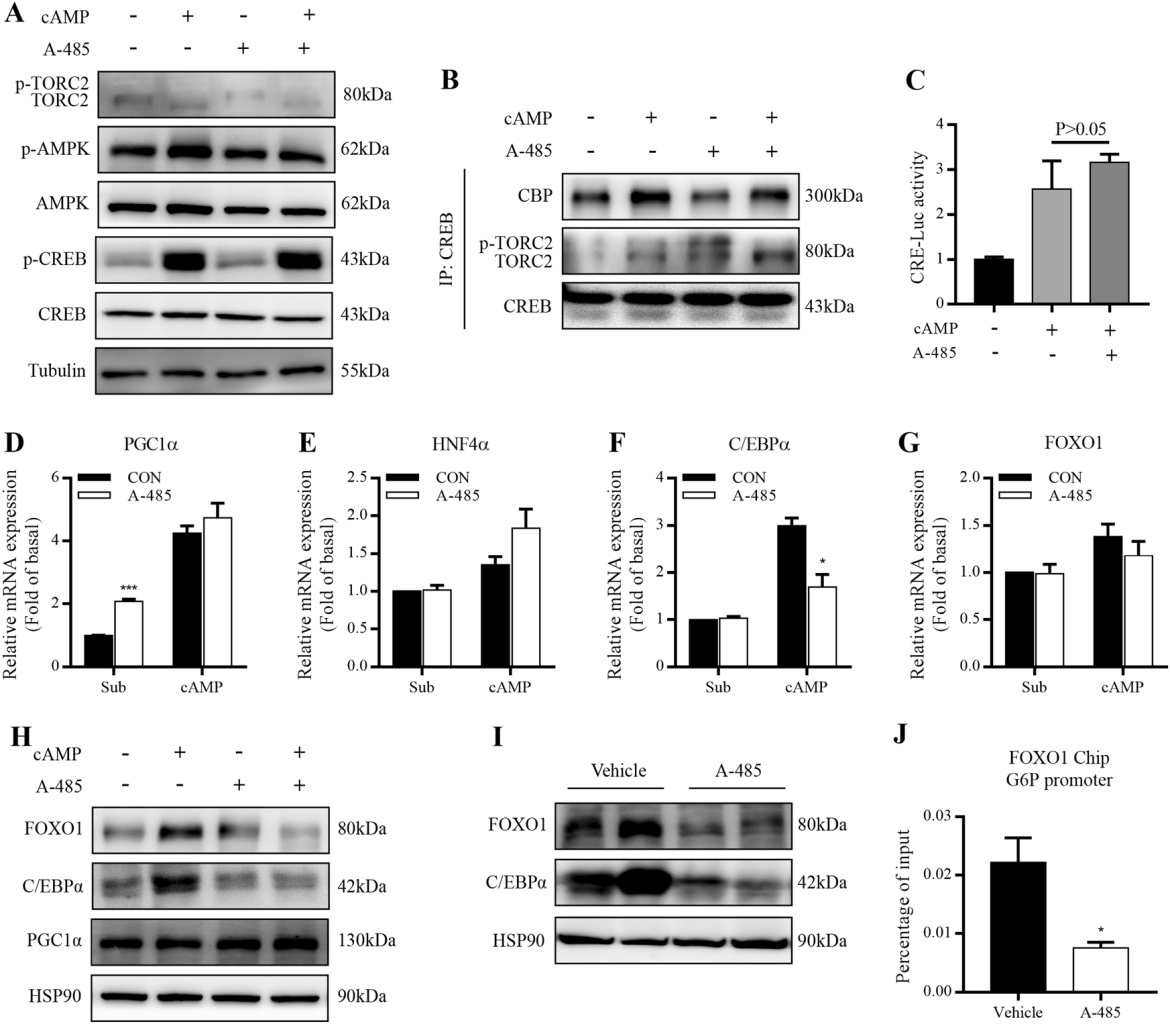
Impact of A-485 on the expressions of gluconeogenesis-related transcriptional factors. **a** Primary mouse hepatocytes were incubated with 3 μM A-485 and 100 μM 8-Br-cAMP for 1 h. The phosphorylation levels of CREB, AMPK, and TORC2 were detected by western blot. **b** The interaction of CREB with CBP and TORC2 was detected in HepG2 cells treated with 3 μM A-485 by coimmunoprecipitation (CoIP). **c** CRE reporter activity was detected in HepG2 cells treated with 3 μM A-485. **d**–**g** mRNA expressions of gluconeogenesis-related transcriptional factors or coactivators in primary mouse hepatocytes treated with 3 μM A-485 and 100 μM 8-Br-cAMP for 8 h. **h** Protein expressions of FOXO1, C/EBPα, and PGC1α in primary mouse hepatocytes treated with 3 μM A-485 and 100 μM 8-Br-cAMP for 12 h. **i** FOXO1 and C/EBPα protein expressions in the liver isolated from vehicle- and A-485-administered mice. **j** ChIP-qPCR analysis of the occupancy of the G6Pc promoter with FOXO1 in the liver isolated from vehicle- and A-485-administered mice. Data are expressed as means ± SEM for three independent experiments. ^*^P < 0.05, ^***^P < 0.001 vs. corresponding control group.

Phosphorylation of transcription factor CREB at Ser133 recruits coactivators such as CBP, p300, and TORC2 to cAMP response element (CRE) containing genes and facilitates gluconeogenesis^24^. It has been demonstrated that phosphorylation of CBP at Ser436 by metformin disrupts CBP binding to CREB, thereby repressing CREB-target gluconeogenic gene expressions and glucose production^25^. Our recent study showed that metformin blocked TORC2 binding to CREB through activating AMPK and inhibiting cAMP-stimulated TORC2 dephosphorylation^26^. Here A-485 did not significantly alter the phosphorylation of CREB or AMPK, nor did it impact TORC2 dephosphorylation in primary hepatocytes (Fig. 6a). Additionally, cAMP-mediated recruitment of CBP to CREB was not changed by A-485 while the recruitment of TORC2 was even enhanced (Fig. 6b). A-485 could not reverse the increased CRE luciferase activity stimulated by 8-Br-cAMP in HepG2 cells (Fig. 6c). cAMP-stimulated mRNA expression of PGC1α, a gluconeogenic coactivator targeted by CREB^27^, was not downregulated by A-485 (Fig. 6d). Therefore, we investigated if other transcription factors were involved in A-485-suppressed gluconeogenesis. No obvious difference was observed for mRNA expression of hepatocyte nuclear factor 4-alpha (Fig. 6e) while cAMP-elicited C/EBPα mRNA expression was attenuated after A-485 treatment (Fig. 6f). Though A-485 failed to alter FOXO1 mRNA expression (Fig. 6g), it antagonized cAMP-elevated FOXO1 protein expression (Fig. 6h). C/EBPα and PGC1α protein expressions exhibited a similar result with their mRNA expressions in the presence of 8-Br-cAMP and A-485 (Fig. 6h). In line with this result, hepatic protein levels of C/EBPα and FOXO1 were lowered in A-485-administered mice compared with control mice (Fig. 6i). The decreased occupancy of G6Pc promoter with FOXO1 was observed in the liver isolated from A-485-administered mice (Fig. 6j). These findings suggest that FOXO1 and C/EBPα are involved in A-485-mediated suppression of gluconeogenesis.

### A-485 deacetylates hepatic FOXO1 and reduces its nuclear abundance

**Fig. 7:**
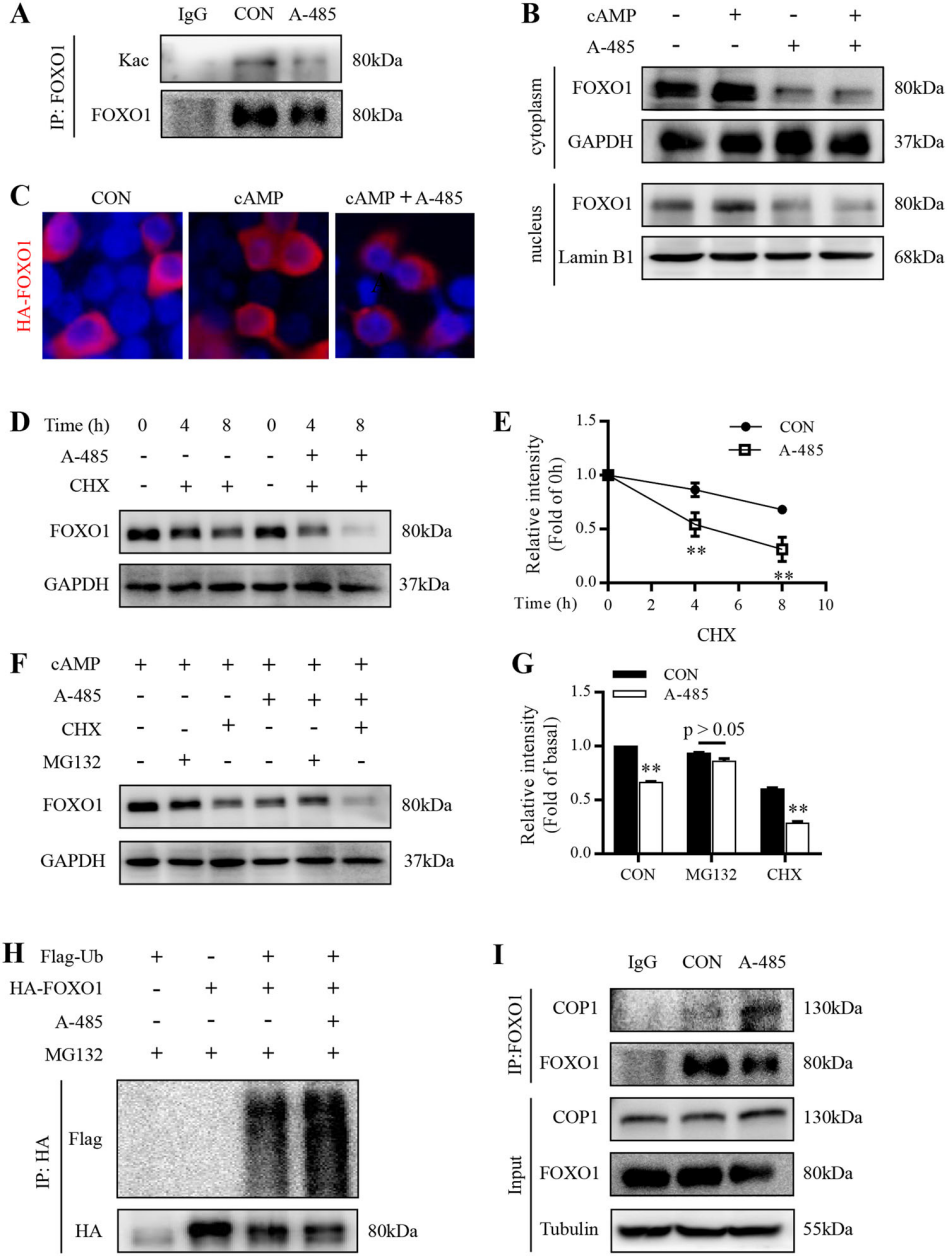
A-485 destabilizes hepatic FOXO1 protein. **a** Endogenous FOXO1 protein were precipitated in HepG2 cells treated with 3 μM A-485 and 100 μM 8-Br-cAMP for 6 h and acetylation of FOXO1 was detected. **b** Cytoplasmic (cyto) and nuclear (nucl) protein levels of FOXO1 in primary mouse hepatocytes treated with 3 μM A-485 and 100 μM 8-Br-cAMP. **c** Immunofluorescence analysis of FOXO1 (red) localization in HEK293T cells transfected with plasmid DNA expressing HA-FOXO1 for 24 h and treated with 3 μM A-485 and 100 μM 8-Br-cAMP for 3 h. **d**, **e** FOXO1 levels in primary mouse hepatocytes treated with 3 μM A-485 and 10 μg/ml cycloheximide (CHX) for the indicated time in the presence of 100 μM 8-Br-cAMP. Signal intensity was quantified by image J software for statistical comparison. **f**, **g** FOXO1 protein level in primary mouse hepatocytes treated with 100 μM 8-Br-cAMP, 3 μM A-485, 10 μg/ml CHX, and 10 μM MG132. Signal intensity was quantified by image J software for statistical comparison. **h** HepG2 cells co-expressed with HA-tagged FOXO1 and Flag-tagged ubiquitin were treated with 3 μM A-485 for 6 h. FOXO1 ubiquitination level was detected. **i** The interaction between FOXO1 and COP1 was detected in HepG2 cells treated with 3 μM A-485. Data are expressed as means ± SEM for three independent experiments. ^**^P < 0.01 vs. corresponding control group.

PTM such as phosphorylation and acetylation is involved in the regulation of FOXO1 activity through primarily affecting its subcellular localization^28^. FOXO1 acetylation could be activated by CBP/p300^29^. Enhanced acetylation promotes FOXO1 nuclear exclusion and inhibits its transcriptional activity^30,31^. As a selective inhibitor of CBP/p300 HAT activity, A-485 did induce the deacetylation of FOXO1 in HepG2 cells (Fig. 7a). Unexpectedly, the protein abundance of FOXO1 was significantly lowered in both cytoplasm and nuclear extracted from primary mouse hepatocytes treated with A-485 (Fig. 7b). FOXO1 was mainly localized in the cytoplasm of 293T cells at basal status and cAMP increased its abundance in nucleus, which was decreased by A-485 treatment (Fig. 7c).

### A-485 destabilizes hepatic FOXO1 protein

A-485 treatment combined with protein synthesis inhibitor CHX dramatically shortened the half-life of endogenous FOXO1 in primary mouse hepatocytes (Fig. 7d, e). In addition, A-485-induced decrease in FOXO1 protein could be readily restored by the proteasome inhibitor MG132 (Fig. 7f, g), suggesting that inhibition of CBP/p300 HAT might target FOXO1 protein to proteasomal degradation. To address this question, HepG2 cells were transfected with HA-FOXO1 together with Flag-Ub expression plasmids. After treatment with MG132, HA-FOXO1 was examined for ubiquitination by Flag antibody. The ubiquitination level of FOXO1 was increased in cells exposed to A-485 (Fig. 7h). There are three types of enzymes involved in the process of ubiquitylation, including ubiquitin-activating enzymes (E1s), ubiquitin-conjugating enzymes (E2s), and ubiquitin-ligases (E3s), among which only E3s determine the target specificity of ubiquitylation reaction^32^. COP1 has been proved as an E3 ligase for FOXO1^33^. In our study, the protein level of COP1 displayed no alteration in primary mouse hepatocytes after A-485 treatment (Fig.S4). However, the interaction between FOXO1 and COP1 was enhanced by A-485 (Fig. 7i). Collectively, these results suggest that A-485 destabilizes FOXO1 protein through promoting its ubiquitination.

## Discussion

CBP/p300 plays a crucial role in the regulation of energy homeostasis through their acetyltransferase activity and their interactions with other transcription factors^34^. Genome-wide association study and network analyses have revealed CBP as the most connected gene in protein–protein interactions in type 2 diabetes^35^. In this current study, A-485, a recently identified first-in-class catalytic inhibitor for HAT domain of CBP/p300, markedly decreased body weight and fat mass as well as improved glucose metabolism in mice under either NCD or HFD condition. Lipogenesis in WAT and liver of mice was markedly suppressed and hepatic gluconeogenesis was decreased after A-485 administration. Thus, the HAT domain of CBP and p300 should be a sensitive drug target for the therapy of obesity-related diseases.

It was reported that CBP^+/−^ mice exhibited lower body weight and fat mass compared with wild-type mice, along with significant smaller size of adipocytes due to the inhibition of lipid accumulation^36^. In another study, CH1 domain deletion of p300 (p300^△CH1/△CH1^) and CBP (CBP^△CH1/△CH1^) mice showed a similar metabolic phenotype, with reduced body weight and WAT^13,36^. Interestingly, the specific inhibition of CBP/p300 HAT domain by A-485 also led to a decrease in body weight and fat mass of mice, which is mainly attributed to the suppression of lipogenesis in WAT. These results indicate that maintaining fat reserves mediated by CBP/p300 is not only limited in transcription factor-binding CH1 domain, HAT domain is also involved. Though the domains of CBP/p300 are significantly shortened constructs and often considered in isolation, they are likely to coordinately regulate the same process. The expressions of lipogenesis-related genes, pan-adipocyte markers, and master regulators of adipogenesis in WAT isolated from A-485-administered mice were all obviously lowered, suggesting that CBP/p300 HAT domain is indispensable for maintaining the function of WAT for energy storage and adipokine secretion.

In addition to WAT, inhibition of lipogenesis by A-485 was also found in hepatocytes. Previous studies indicate an intriguing connection between elevated acetylation of transcription factors such as FXR, SREBP-1c, and ChREBP, and increased lipogenic gene expressions^20–22^, which provides a therapeutic strategy for nonalcoholic fatty liver disease. p300 has been proved to acetylate hepatic ChREBP on lys672 and increase its transcriptional activity, but increased acetylation mediated by p300 fails to affect ChREBP expression^20^. Surprisingly, we found that A-485 directly decreased the expression of hepatic ChREBP. It is possible that the inhibition of another acetyltransferase CBP by A-485 explains the different result. In spite of the high sequence homology between CBP and p300, they may exert distinct effects on the same target. In the liver of CBP^+/−^ mice, tissue triglyceride content was markedly reduced^37^. Apparently, a reduction in hepatic lipogenic gene expression mediated by disrupting HAT domain of CBP/p300 contributes to decreased accumulation of lipid in liver.

Besides lipid metabolism, CBP and p300 are crucial regulators of glucose homeostasis^34^. CBP is a CREB binding protein that interacts with the CREB KID domain^25^. Glucagon stimulates hepatic gluconeogenesis via promoting the formation of transcriptional CREB–CBP–TORC2 complex on a CRE site of target genes^38^. p300 overexpression led to glucose intolerance in mice, with increased expressions of PEPCK and G6Pc^20^. In this current study, the decreased fasting blood glucose level in A-485-administered mice agreed well with the enhanced pyruvate tolerance and reduced hepatic G6pc expression, inconsistent with previously published results on CBP^KIX/KIX^ and CBP^△CH1/△CH1^ mice^13,24^. Thus, it is CBP/p300 HAT domain that dominates its regulatory role in hepatic gluconeogenesis, which is further confirmed by in vitro experiments. In addition, metformin reduces gluconeogenic gene expression via inducing the phosphorylation of CBP and TORC2, triggering the dissociation of the CREB–CBP–TORC2 complex^25,26^. Our study revealed a synergistic effect of A-485 and metformin on the inhibition of gluconeogenesis in primary hepatocytes. Moreover, the interaction of CREB–CBP–TORC2 complex was not altered by A-485. These results suggest that A-485 represses hepatic gluconeogenesis through a pathway distinct from metformin.

FOXOs belong to an evolutionarily conserved mammalian forkhead family of transcription factors, which regulate cell differentiation, metabolism, proliferation, and survival^38,39^. Among FOXO family members, FOXO1 is not only tightly linked to hepatic gluconeogenesis^40^, but also enhances lipogenesis and liver steatosis^41,42^. Glucose is used for de novo lipogenesis in the liver, in which FOXO1 might be involved^43^. In our study, A-485 decreased hepatic FOXO1 protein level. FOXO1 is traditionally regarded as an important component of insulin signaling cascades in suppressing gluconeogenesis^44,45^. A recent study demonstrates a novel regulatory mechanism of FOXO1 by glucagon, which promotes FOXO1 stability and nuclear retention via cAMP/PKA-dependent phosphorylation of FOXO1 at Ser276^18^. In this study, A-485 blocked cAMP-increased abundance of FOXO1 protein in nucleus and enhanced proteasome-mediated FOXO1 ubiquitination in primary hepatocytes. FOXO1 acetylation is activated by CBP at Lys242, Lys245, Lys262^29^. In β cells, acetylated FOXO1 is retained in the nucleus and deacetylation of FOXO1 by SIRT1 accelerates FOXO1 degradation^46^. However, a number of literatures hold the view that deacetylation of hepatic FOXO1 promotes its nuclear retention, thus increasing its activity for gluconeogenesis^30,31,47^. In our study, A-485-mediated deacetylation of FOXO1 promoted its degradation in hepatocytes through ubiquitination, leading to the inhibition of gluconeogenesis.

In summary, A-485 disrupted lipogenesis in WAT and liver as well as suppressed hepatic gluconeogenesis in C57BL/6 mice, leading to the loss of body weight and improvement of glucose metabolism. A-485-mediated degradation of FOXO1 protein through a proteasome-dependent pathway is involved in the suppression of gluconeogenesis and lipogenesis. Therefore, the specific inhibition of CBP/p300 HAT will offer promise for a novel therapeutic approach of metabolic diseases.

## Supplementary information

supplemental data legend

supplement 1

supplement 2

supplement 3

supplement 4

Supplementary Table 1

Supplementary Table 2

Supplementary Table 3
