# Supplementary material for: Selective inhibition of CBP/p300 HAT by A-485 results in suppression of lipogenesis and hepatic gluconeogenesis
Source: Cell Death Dis. 2020 Sep 11;11(9):745. doi: 10.1038/s41419-020-02960-6 (PMC7486386; doi:10.1038/s41419-020-02960-6)
Supplement: Supplementary file 1 — supplemental data legend [file 41419_2020_2960_MOESM1_ESM.docx]

**Supplementary figure legend**

**Fig.S1.** **Supplemental metabolic phenotype of A-485-adimistrated mice.** **(a**) Effect of A-485 on food intake. (**b**-**d**) The oxygen consumption (VO_2_), carbon dioxide (VCO_2_) production, and respiratory exchange ratios (RER) during 48 h. (**e**-**f**) Plasma total cholesterol (TC) and triglyceride (TG) after a 16h fasting. Data are expressed as means ± SEM (*n*=5–9).

**Fig.S2. Inhibited expression of lipogenesis-related genes in the iWAT by A-485. (a**) Morphology of the iWAT and eWAT from NCD-fed mice treated with vehicle and A-485. **(b** and **c**) mRNA expressions of lipogenesis-related genes in the iWAT from NCD- and HFD-fed mice. (**d**) mRNA expression of UCP1 in the WAT isolated from NCD-fed mice treated with A-485 and vehicle. (**e** and **f**) Adiponectin, FABP4, and LPL mRNA expressions in the iWAT from NCD-mice and HFD-mice. (**g** and **h**) PPARγ and C/EBPα mRNA expressions in the iWAT from NCD- and HFD-fed mice. Data are expressed as means ± SEM (*n*=5–9). ^*^*P*< 0.05, ^**^*P*< 0.01 *vs* corresponding control group.

**Fig.S3. A-485 inhibits the expression of lipogenesis-related genes in differentiated primary adipocytes.** Stromal vascular (SV) cells were isolated from iWAT of C57BL/6 mice for white adipocyte differentiation. Fully differentiated mature adipocytes were treated with A485 for 24 h since day 8 of differentiation. **(a**) mRNA expressions of lipogenesis-related genes detected by RT-qPCR. **(b**) Western blot analysis of FAS and ACC protein levels. (**c**) Adiponectin, FABP4, and LPL mRNA expressions. (**d** and **e**) PPARγ and C/EBPα mRNA and protein expressions. Data are expressed as means ± SEM (*n*=5). ^*^*P*< 0.05, ^**^*P*< 0.01 *vs* control group.

**Fig.S4. A-485 does not impact the protein expression of COP1 in primary mouse hepatocytes.**  Primary mouse hepatocytes were incubated with 3 μM A-485 and 100 μM 8-Br-cAMP for 12 h. The protein level of COP1 was detected by Western blot.
