# Supplementary figures and images for: Selective inhibition of CBP/p300 HAT by A-485 results in suppression of lipogenesis and hepatic gluconeogenesis
Source: Cell Death Dis. 2020 Sep 11;11(9):745. doi: 10.1038/s41419-020-02960-6 (PMC7486386; doi:10.1038/s41419-020-02960-6)

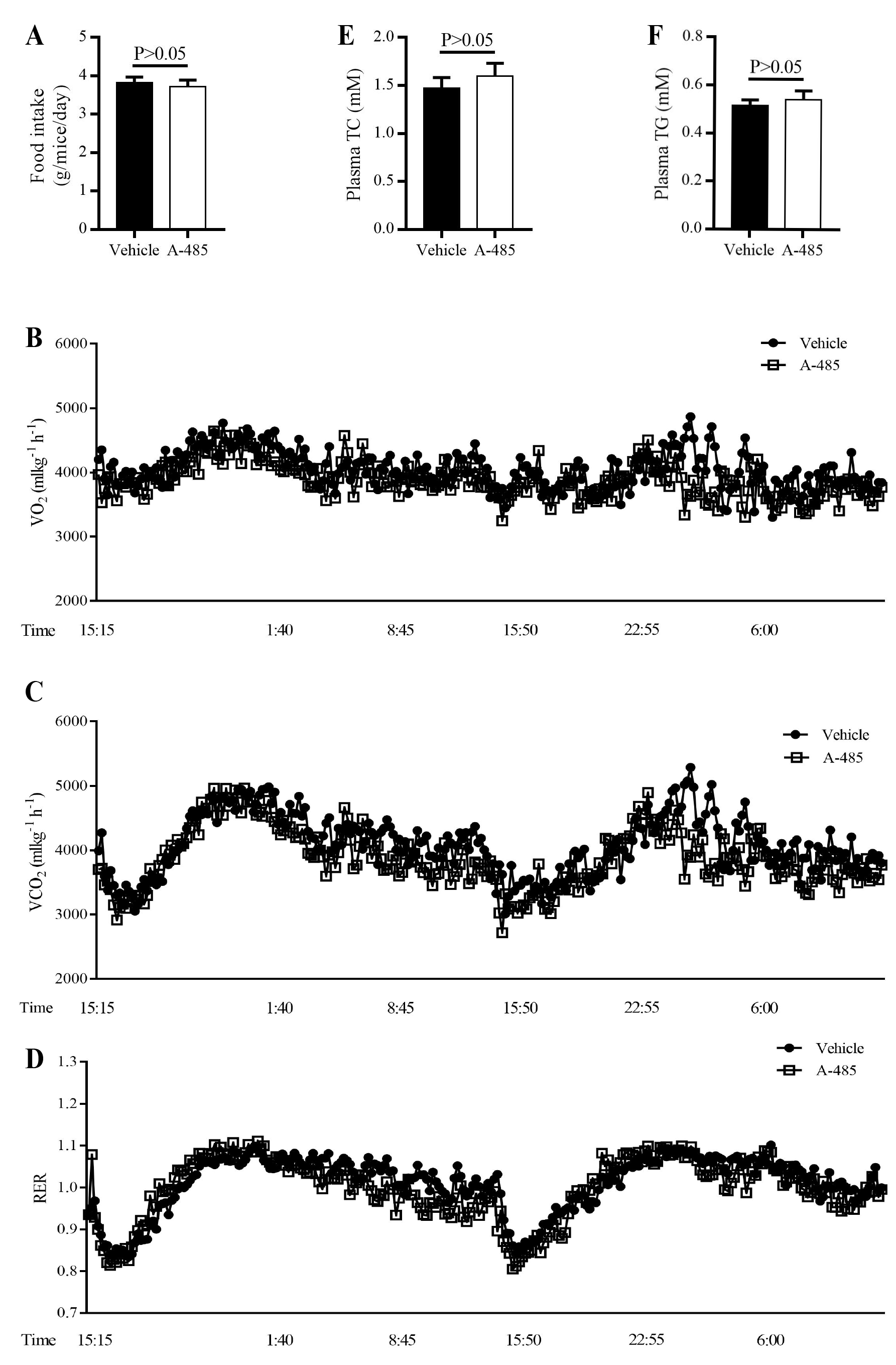

Supplement: Supplementary file 2 — supplement 1 [file 41419_2020_2960_MOESM2_ESM.tif]

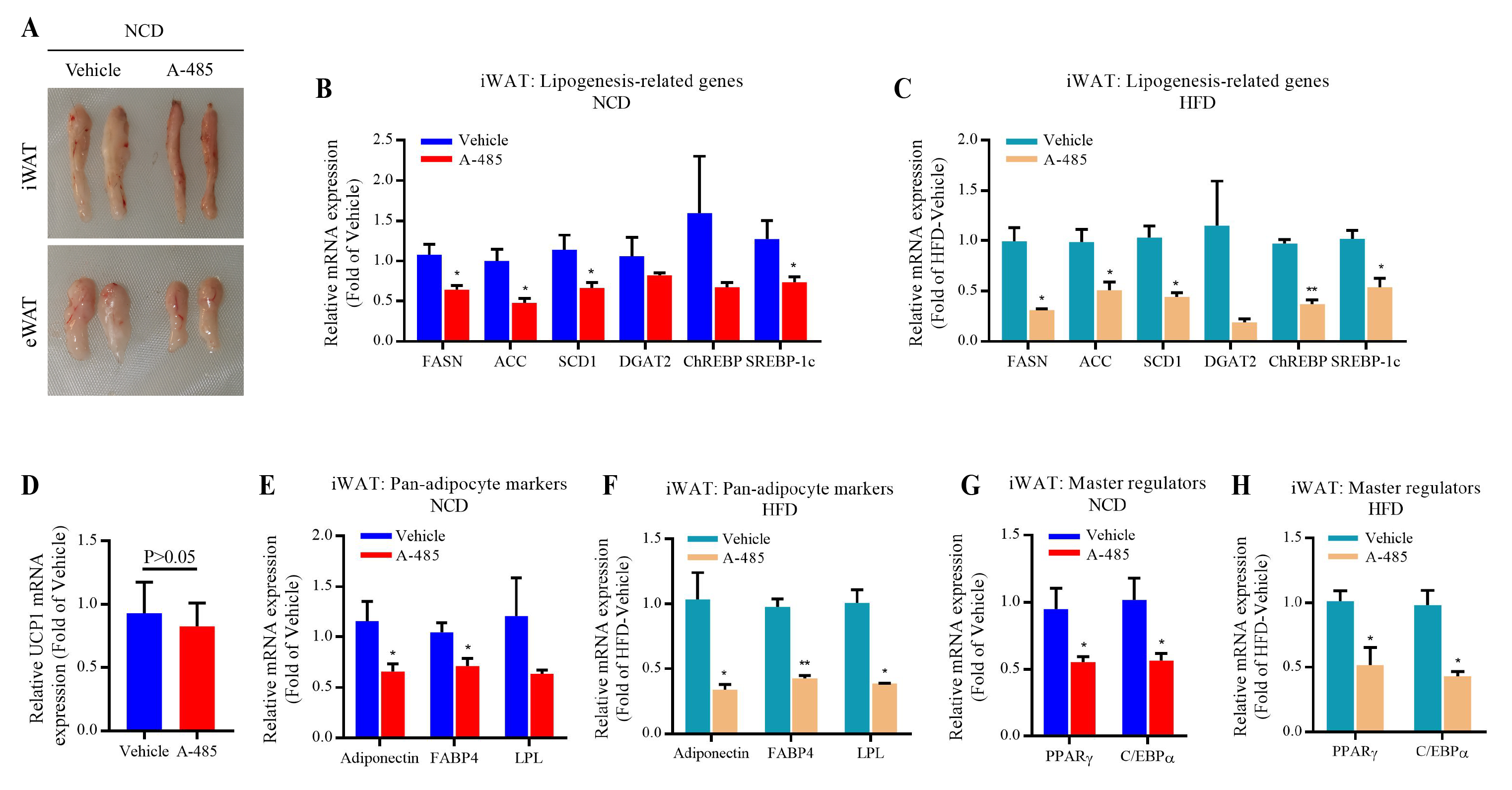

Supplement: Supplementary file 3 — supplement 2 [file 41419_2020_2960_MOESM3_ESM.tif]

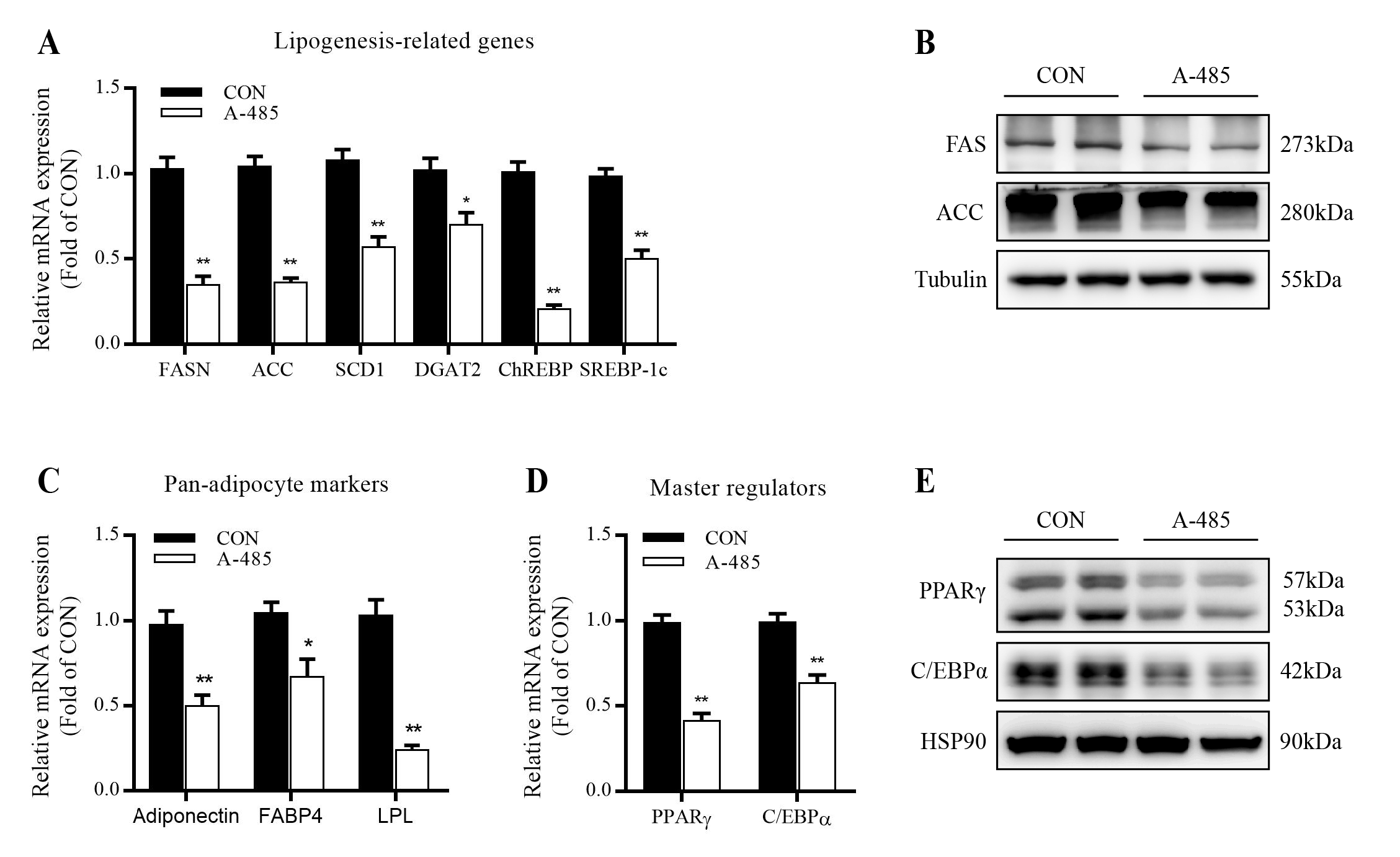

Supplement: Supplementary file 4 — supplement 3 [file 41419_2020_2960_MOESM4_ESM.tif]

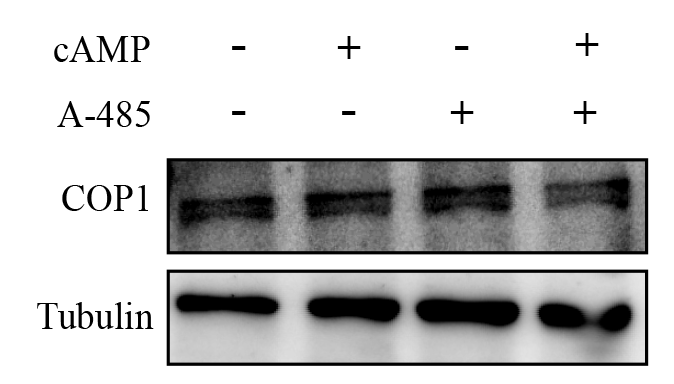

Supplement: Supplementary file 5 — supplement 4 [file 41419_2020_2960_MOESM5_ESM.tif]

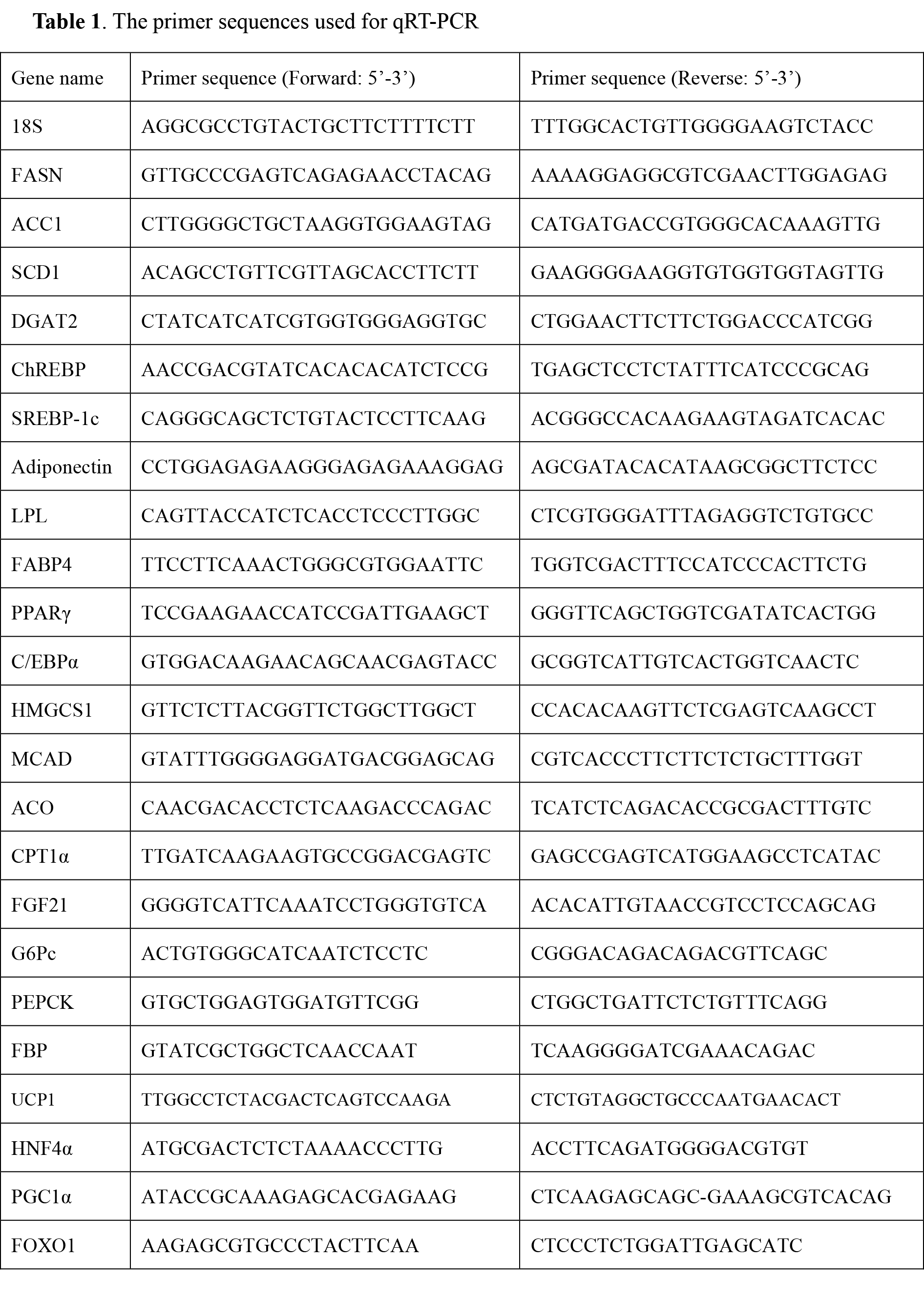

Supplement: Supplementary file 6 — Supplementary Table 1 [file 41419_2020_2960_MOESM6_ESM.tif]

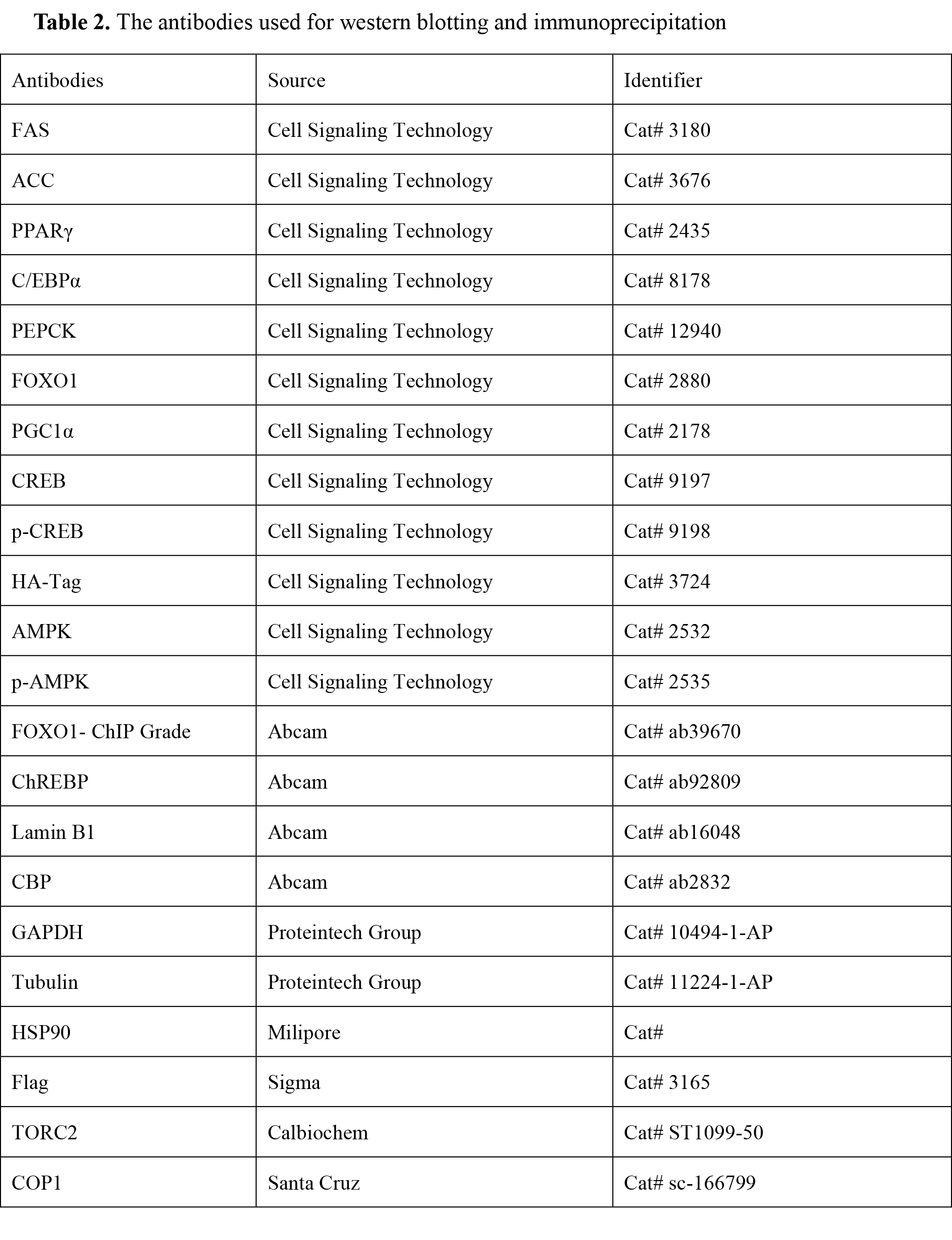

Supplement: Supplementary file 7 — Supplementary Table 2 [file 41419_2020_2960_MOESM7_ESM.tif]

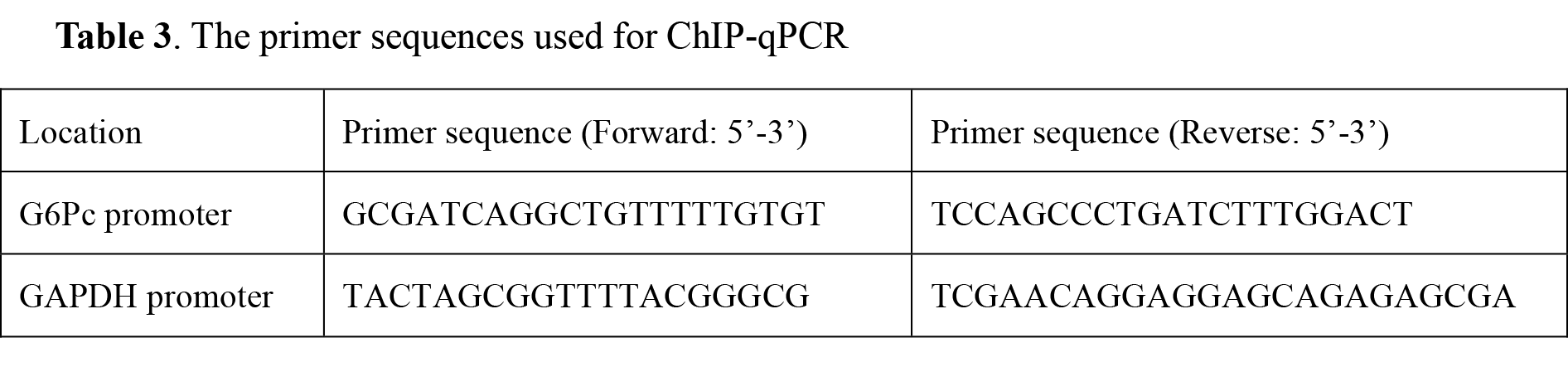

Supplement: Supplementary file 8 — Supplementary Table 3 [file 41419_2020_2960_MOESM8_ESM.tif]
